# *MbMYBC1*, a *M. baccata* MYB transcription factor, contribute to cold and drought stress tolerance in transgenic *Arabidopsis*

**DOI:** 10.3389/fpls.2023.1141446

**Published:** 2023-02-16

**Authors:** Wanda Liu, Tianhe Wang, Yu Wang, Xiaoqi Liang, Jilong Han, Deguo Han

**Affiliations:** ^1^ Horticulture Branch, Heilongjiang Academy of Agricultural Sciences, Harbin, Heilongjiang, China; ^2^ Key Laboratory of Biology and Genetic Improvement of Horticultural Crops (Northeast Region), Ministry of Agriculture and Rural Affairs/National-Local Joint Engineering Research Center for Development and Utilization of Small Fruits in Cold Regions/College of Horticulture & Landscape Architecture, Northeast Agricultural University, Harbin, China

**Keywords:** *Malus baccata* (L.) Borkh, *MbMYBC1*, transgenic technology, cold stress, drought stress

## Abstract

Cold and drought stress considerably suppress the development of plants. In this study, a new MYB (v-myb avian myeloblastosis viral)TF gene, *MbMYBC1*, was isolated from the *M. baccata* and located in nucleus. *MbMYBC1* has a positive response to low temperature and drought stress. After being introduced into *Arabidopsis thaliana*, the physiological indicators of transgenic *Arabidopsis* had corresponding changes under these two stresses, the activities of catalase (CAT), peroxidase (POD) and superoxide dismutase (SOD) increased, electrolyte leakage rate (EL) and the content of proline increased, but the content of chlorophyll decreased. In addition, its overexpression can also activate the downstream expression of *AtDREB1A*, *AtCOR15a*, *AtERD10B* and *AtCOR47* related to cold stress and *AtSnRK2.4*, *AtRD29A*, *AtSOD1*and *AtP5CS1* related to drought stress. Based on these results, we speculate that *MbMYBC1* can respond to cold and hydropenia signals, and can be used in transgenic technology to improve plant tolerance to low temperature and drought stress.

## Introduction

Various uncertain factors in the natural environment seriously restrict the development of apple industry and cause serious damage to it, especially low temperature, drought and osmotic stress (Chinnusamy et al., 2006; Liu et al., 2023). When sensing external stress signals, plants will undergo a variety of changes, including physiological and biochemical levels, to reduce their own damage (Han et al., 2018a). Therefore, according to these changes, the damage degree of plants can be roughly known. Stress destroys the integrity of organelles and enhances the permeability of membranes, the more serious the damage of cell membranes, the greater the electrolyte leakage rate (EL) (Blum, 1983). It will also accelerate the degradation of chlorophyll and reduce the photosynthetic rate. During the normal growth of plants, the content of ROS in cells is maintained in a normal state, while stress will destroy the balance of its production and removal (Nandhini et al., 2022). Too much ROS is easy to cause oxidative stress if it is not cleared in time. Superoxide dismutase (SOD), peroxidase (POD) and catalase (CAT) can effectively remove ROS, and their activities can be used to reflect the antioxidant level of plants (Dogru and Çakirlar, 2020; Liang et al., 2022a). Plants can resist the damage of organelles, proteins and cell membranes caused by external stimuli by regulating the accumulation of osmoregulation substances (Gerona et al., 2019; Yao et al., 2022a). In addition, the phenotype of plants will also change significantly.

In order to survive under various environmental conditions, plants have formed a series of stress response mechanisms. It has been proved that transcription factor (TF) can effectively help plants resist adversity (Jiang and Deyholos, 2009; Han et al., 2020a). According to the number of domain repeats, MYB TFs are divided into four subfamilies: 1R-MYB, 2R-MYB, 3R-MYB and 4R-MYB (Li W. et al., 2022). Among them, 2R-MYB TF is the most common in plants and is characterized in many plants (Li et al., 2015). For example, there are 126, 108, 157 and 222 2R-MYB genes in *Arabidopsis* (Li et al., 2016), grape (Matus et al., 2008), corn (Du et al., 2012) and apple (Cao et al., 2013) respectively. MYB is not only widely involved in various metabolic activities, material synthesis and other biological processes of plants, but also can protect plants from abiotic stresses such as high salt, low temperature, heat and drought (Lin et al., 2021). Studies have shown that soybean *GmMYB118* can make plants more adaptable to drought and salinity by regulating osmotic pressure and oxidant and inducing the expression of stress-related genes (Du et al., 2018). In tomato, the overexpression of *SlMYB102* can enhance the resistance of plants to cold, and may also participate in CBF and proline synthesis pathway to further improve the adaptability of tomato to stress (Wang et al., 2020).

The process of plant response to stress often involves the participation of multiple genes through multiple channels. The process of plant resistance is generally divided into two steps, first sensing the stress signal and then transmitting it; second, activating the expression of various related genes. CBF/DREB is rapidly induced by low temperature in the cold response pathway and combines with the C-repeat/dehydration response region in the cold response (*COR*) gene promoter to improve cold resistance (Yamaguchi-Shinozaki and Shinozaki, 1994; Li X. et al., 2022). Drought stress response protein can improve the viability of plants under water shortage conditions through ABA-dependent and ABA-independent pathways (Wassie et al., 2023). Studies have proved that there are ABA response elements in promoters of many genes, such as *RAB18*, *RD22*, *RD29* (Kazuko and Kazuo, 1993; Latchman, 1997). The expression of proline biosynthesis gene *P5CS1* will increase under drought stress, its transcription level can reflect the proline accumulation under drought stress. Moreover, *P5CS1* regulation does not depend on ABA signal transduction pathway (Furlan et al., 2020).

*Malus baccata* (L.) Borkh (*M. baccata*) is a commonly used grafting rootstock in apple cultivation (Ren et al., 2017), and the cold tolerance and drought tolerance of rootstock have a direct impact on the environmental adaptability of apple, so the study on the stress tolerance of rootstock is of great significance in the process of apple breeding. However, there are few studies on MYB in *M. baccata*. In this study, we cloned and identified the expression of *MbMYBC1* and stress related genes in *A. thaliana*, and preliminarily clarified the cold and drought resistance mechanism of mountain stator. It has promoted the development of the breeding of *M. baccata* stress resistant varieties as rootstocks, laid a foundation for the research on the molecular mechanism of cold and drought resistance of apples, and is conducive to the selection of *Malus plants* breeding genes.

## Materials and methods

### Cultivation and treatment of *M. baccata*

*M. baccata* tissue culture plantlets was put on Murashige and Skoog (MS) medium containing 0.55 mg/L cytokinin (6-BA) and 0.6 mg/L indole butyric acid (IBA) for rapid propagation. After one month, selected robust tissue culture plantlets and transfered them to rooting medium (1.2 mg/L IBA) (Yao et al., 2022b). After rooting, seedlings were put into Hoagland hydroponic culture solution for growth. The room temperature and humidity of tissue culture were maintained at about 25°C and 80%. Changed the hydroponic culture solution regularly. When 7-8 fully developed mature leaves and new strong roots grow out of the hydroponic seedlings, seedlings with roughly the same growth conditions were selected and grouped, 10 seedlings in each group, a total of 5 groups, one of which was the control group without any treatment. The other 4 groups were treated as follows respectively, and the hydroponic seedlings were placed in the 4°C tissue culture room for low temperature stress; The Hoagland hydroponic culture solution with 200 mM NaCl was used for salt stress; Hoagland hydroponic medium containing 20% PEG6000 was used for water stress; The hydroponic seedlings were put into the tissue culture room at 37°C for high temperature stress. The young leaves, mature leaves, roots and stems of all seedlings were sampled after treatment for 0, 1, 3, 6, 9 and 12 h, and stored at -80°C after liquid nitrogen quick freezing (Han et al., 2018b).

### Cloning and qRT-PCR expression analysis of *MbMYBC1*

The total RNA was extracted from the roots, stems and leaves (young leaves and mature leaves) of *M. baccata* seedlings with EasyPure plant RNA kit (TransGen Biotech, Beijing, China). Then used Trans Script^®^ First-Strand cDNA Synthesis Super Mix (transgen, Beijing) to synthesize the first strand of cDNA. According to the CDs region of *MbMYBC1*, two pairs of specific primers were designed with Primer 5.0 software. After synthesizing the primers, *MbMYBC1*-F and *MbMYBC1*-R were obtained (**Supplementary Table S1**). The full length of the target gene was obtained by PCR using the first strand cDNA of apple as the template. After detection and purification by agarose gel electrophoresis, the target gene was linked to the cloning vector using pEASY-T1 cloning kit (TransGen Biotech, Beijing, China) and sequenced (BGI, Beijing) (Han et al., 2020a).

To analyze the expression of *MbMYBC1*, it was detected by qRT-PCR. *MbActin* was used as a control, which can be stably expressed without being affected by conditions (Modesto et al., 2013), and the gene is amplified from *M. baccata* tissues. The primers *Actin*-F and *Actin*-R were designed according to the sequences published in the GenBank database (**Supplementary Table S1**). From the partial sequences obtained in this study, *MbMYBC1* primer for qRT-PCR was designed, *MbMYBC1*-qF/qR (**Supplementary Table S1**). The qPCR reaction system was shown in **Supplementary Table S2**, and its reaction process was as follows: pre denaturation at 95°C for 5 min, denaturation for 5 s, annealing at 60°C for 1 min, and extension at 72°C for 1 min. Carried out 35 cycles and continued to extend at 72°C for 5 min (Han et al., 2013; Han et al., 2015; Assal and Lin, 2021). The 2^−ΔΔCT^ method was used to analyze the relative transcriptional level data of the target gene (Li Y. et al., 2022).

### Bioinformatics analysis of the *MbMYBC1*

On ExPASy website (https://web.expasy.org/protparam/), the primary structure and various physical and chemical properties of the target protein were predicted (Han et al., 2020b). The domain and tertiary structure of MbMYBC1 protein were predicted on SMART and SWISS-MODEL websites respectively. The sequences of MbMYBC1 were blasted in NCBI database, MYB sequences of several other species with high sequence similarity was select. Compared these sequences with DNAMAN 5.2, and then constructed phylogenetic tree through MEGA7 neighbor connection method. These amino acid sequences were PbMYBC1 (*Pyrus bretschneideri*, XP_048430460.1), MaMYBC1 (*Mercurialis annua*, XP_050229005.1), CiMYBC1 (*Carya illinoinensis*, XP_042940585.1), JcMYBC1, (*Jatropha curcas*, XP_012088642.1), JrMYBC1 (*Juglans regia*, XP_035544691.1), CsMYBC1 (*Cannabis sativa*, XP_021654469.1), MsMYBC1 (*Malus sylvestris*, XP_050115747.1), EgMYBC1(*Eucalyptus grandis*, XP_010036386.2), PaMYBC1 (*Prunus avium*, XP_021808479.1), AtMYBC1 (*Arabidopsis thaliana*, AT2G40970).

### Subcellular localization analysis of the *MbMYBC1* protein

The enzyme digestion sites of *BamHI* and *SalI* were selected on pSAT6-GFP-N1 vector, and MbMYBC1-ORF was cloned between these two sites to obtain MbMYBC1-GFP fusion protein. The modified red shift green fluorescent protein (GFP) was contained between these two sites. The MbMYBC1-GFP plasmid was injected into the tobacco epidermal cells from the lower epidermis of the leaves using the Agrobacterium tumefaciens injection method, and the empty 35S:: GFP plasmid as the control was also transferred into the leaves (Su et al., 2022). The expression of MbMYBC1-GFP was observed under confocal microscope to determine its location.

### Acquisition of transgenic *A. thaliana*

The 5’ and 3’ ends of *MbMYBC1* were linked to the restriction sites of *BamHI* and *SalI* by PCR to construct the overexpression vector of *Arabidopsis* transformation. *BamHI* and *SalI* digested PCAM3011 and PCR products and replaced them with GUS gene to connect them. PCAMBIA2300-*MbMYBC1* was transferred into GV3101, and then the *MbMYBC1* gene was introduced into Colombian ecotype *A. thaliana* Co1-0 through inflorescence mediation. After screening by 1/2 MS medium containing 50 mg/L kanamycin, the successfully transformed T_3_ generation *A. thaliana* was used for subsequent research.

### Stress treatment and physiological index determination of transgenic *A. thaliana*

All *A. thaliana* (WT, UL, L1, L4, L5) seeds were sowed on the culture medium, and were transferred to a flowerpot containing the same amount of vermiculite and nutrient soil 15 d later, with a tray at the bottom of the flowerpot. When *A. thaliana* had 8-12 new leaves, they were divided into 3 groups: one group grew normally, one group was treated at -4°C for 12 h, and the other group were stopped watering within 7 d. The growth of plants was resumed for 3 d under normal conditions after stress treatment. The surface changes of each line were observed and the changes of its survival rate and related physiological and biochemical indexes were measured. Three biological replicates and three technical replicates were performed for each sample.

The EL of the sample was determined according to the method of Campos et al. (2003), determination of chlorophyll content by grinding centrifugation with 95% ethanol (Wang et al., 2022). The content of proline in samples was determined by standard curve method according to the description of Ou et al. (2015). The activity of SOD, CAT and POD was determined with the kit of Nanjing Jiancheng Bioengineering Research Institute (Nanjing, China).

### Analysis of *MbMYBC1* downstream gene expression

The mRNA of *A. thaliana* grown under 3 conditions was extracted and reverse transcribed to obtain the first strand of cDNA which was used as a template. Using *Actin* as internal reference gene, several important downstream stress related genes of MYB were detected by qPCR. These genes were *AtDREB1A* (Lu et al., 2017), *AtCOR15a* (Steponkus et al., 1998), *AtERD10B* (Du and Li, 2019), *AtCOR47* (Song et al., 2021), *AtSnRK2.4* (Nakashima and Yamaguchi-Shinozaki, 2013), *AtRD29A* (Wu et al., 2022), *AtSOD1* (Li J. et al., 2022) and *AtP5CS1* (Chen et al., 2015). The reaction procedure and system of qPCR were the same as above methods.

### Statistical analysis

SPSS 21.0 software (IBM, Chicago, Illinois, USA) was used for one-way analysis of variables. All test data were the average values obtained after 3 repetitions, and their standard errors (± SE) were calculated. The statistical difference was referred to as significant * p ≤ 0.05 and **p ≤ 0.01.

## Results

### Bioinformatics analysis of *MbMYBC1* gene

The results obtained by ExPASy analysis (**Supplementary Figure S1**) showed that the open reading frame (ORF) of MbMYBC1 was 948 bp, 315 amino acids (aa) were encoded by MbMYBC1, and the largest proportion of these 18 aa was Pro (13.6%), Ala (10.5%), Ser (8.3%), the theoretical molecular weight (MW) was 34.048 kDa. The aliphatic index of MbMYBC1 protein was 64.22, the theoretical isoelectric point (pI) was 6.34, and the instability index was 62.46. Therefore, it was hydrophilic and unstable.

**Figure 1:**
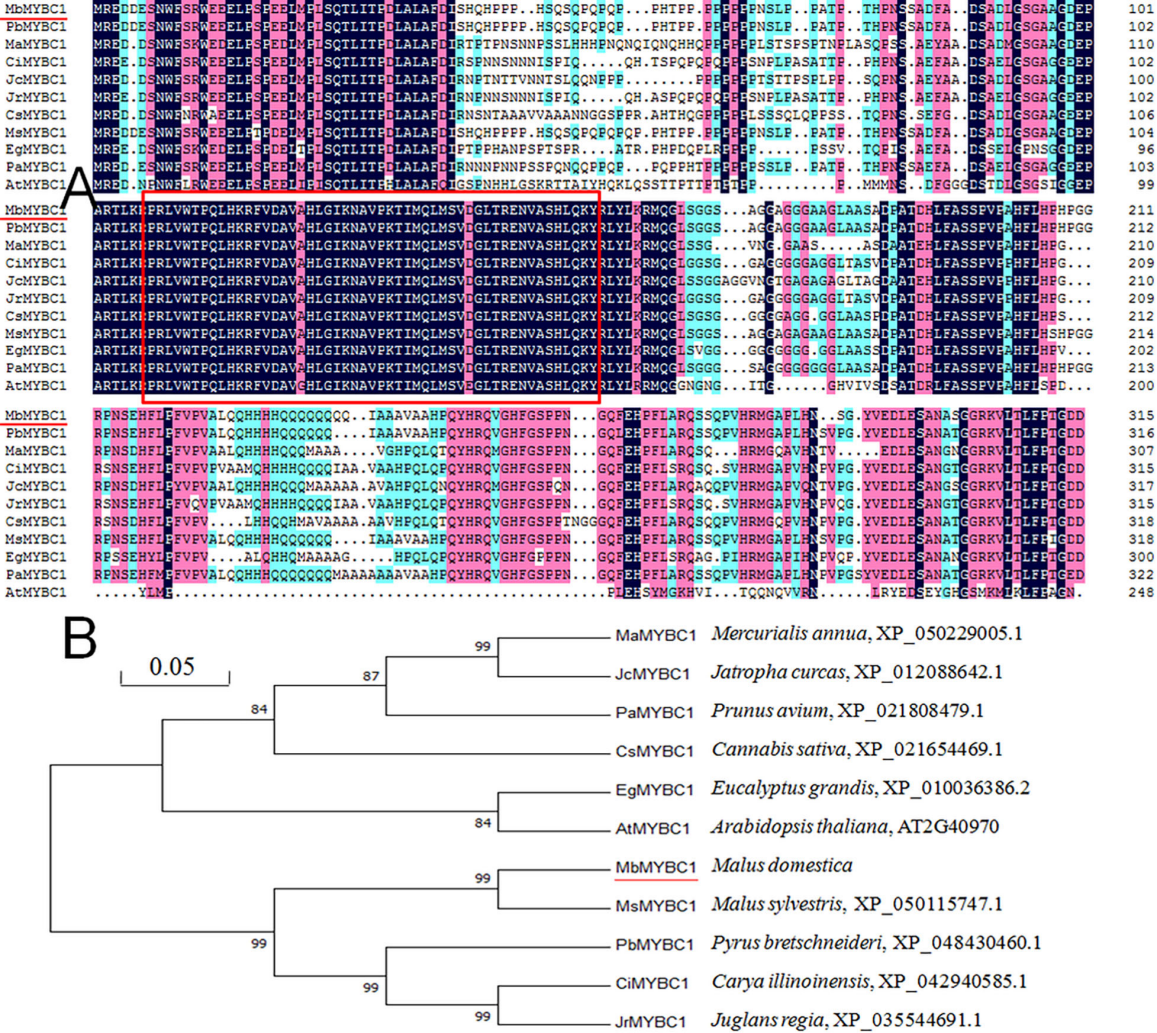
Similarity analysis of amino acid sequences between MbMYBC1 and 10 other MYBs **(A)** and comparison of genetic relationships **(B)**. The red underline is the target protein, and the part in the box is the MYB structural domain.

**Figure 2:**
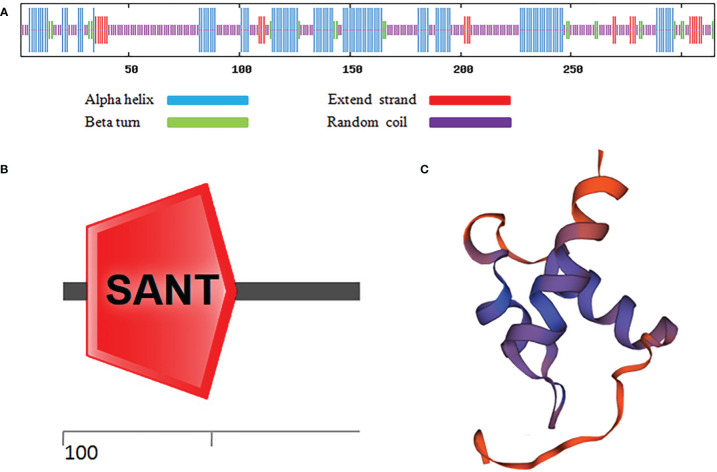
Prediction of Secondary and Tertiary Structure of MbMYBC1 Protein. **(A)** Predicted protein secondary structure; **(B)** predicted protein domains; **(C)** predicted tertiary structure.

As shown in **Figure 1A**, after sequence alignment with 10 other MYBC1 proteins with high homology, it was found that MbMYBC1 protein contained a unique conservative domain of MYB-TF, indicating that it was a MYB protein. The evolutionary tree showed that MbMYBC1 and MsMYBC1 were clustered in the first cluster of the tree, with the closest genetic relationship and the highest homology (**Figure 1B**). **Figure 2A** showed the analysis results of the secondary structure of MbMYBC1 protein. It can be seen that it contained 33.9% α-Helix, 6.35% β-coil, 7.3% extended strand and 52.38% random coil (**Figure 2B**). Its tertiary structure conformed to the predicted secondary structure, which contained HTH region (**Figure 2C**). These results indicated that MbMYBC1 was a member of 3R-MYB subgroup.

### Subcellular localization of *MbMYBC1* proteins

**Figure 3:**
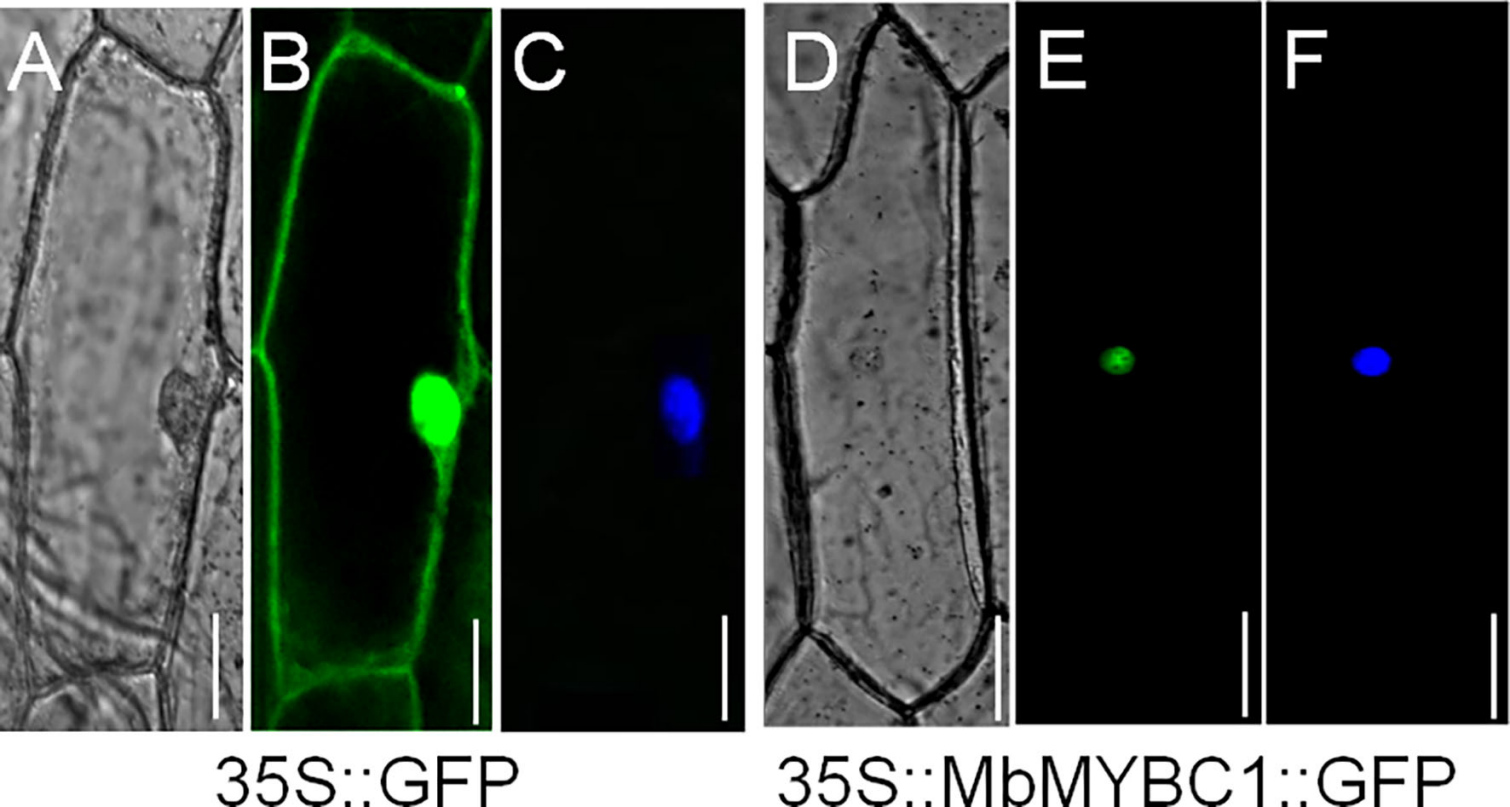
Subcellular localization of MbMYBC1 protein. **(A, D)** Bright-field images, **(B, E)** GFP fluorescence, **(C, F)** the effects after DAPI dyeing. Bar = 50 μm.

**Figure 3** showed the distribution of 35s::MbMYBC1::GFP under the confocal microscope. The green fluorescence of 35s::GFP protein was distributed in the whole cell (**Figure 3B**), while the green fluorescence of 35s::MbMYBC1::GFP fusion protein was only distributed in the nucleus (**Figure 3D**). The nuclear position was finally determined after staining with 4 ‘, 6-diamino-2-phenylindole (DAPI) (**Figure 3E**). That was to say, it can be preliminarily determined that MbMYBC1 was a nuclear protein.

### Expression level of *MbMYBC1* gene

**Figure 4:**
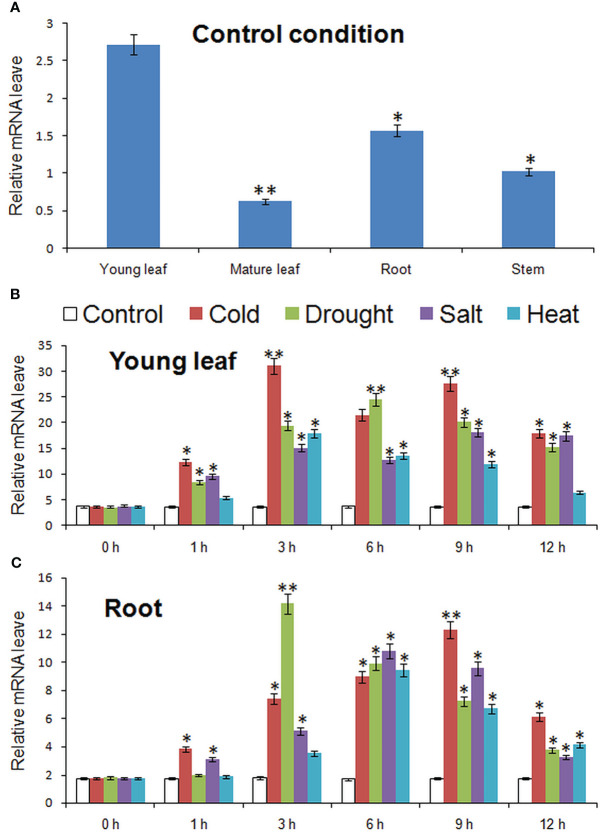
Results of qPCR analysis of *MbMYBC1*. **(A)** The expression of *MbMYBC1* in different parts of in *M. baccata*; The expression of *MbMYBC1* in new leaves **(B)** and roots **(C)** under different stresses. Compared with the Control, the asterisks above the column indicate significant difference and extremely significant difference (*, *p ≤*0.05; **, p ≤ 0.01).

**Figure 4** showed the expression of *MbMYBC1* in various tissues and the change of expression level in different time periods under various stresses. The results in **Figure 4A** showed that although *MbMYBC1* can be expressed in these organs (root, stem and leaves), there were significant differences in the amount of expression. The expression of *MbMYBC1* decreased in turn in young leaves, roots, stems and mature leaves. The expression of *MbMYBC1* in new leaves was 6.78 fold higher than that in mature leaves.

The expression of *MbMYBC1* in young leaves and roots increased first and then decreased with the stress time. **Figure 4B** showed this result, in young leaves, the stress that made the expression of *MbMYBC1* gene reached the peak first was the low and high temperature conditions (3h), and the expression levels under these two conditions were 8.41, 5.89 fold higher than those under normal conditions respectively. Under salt and drought stress, the expression level at 9 and 6 hours was the highest, respectively, which was 4.79 and 6.82 fold of those of the control. But in the root, the peak of expression was reached first under drought (3h), 7.88 times of that of untreated. Under high salt and high temperature conditions, the expression level was the highest at 6 h, 6.22 and 5.34 fold higher than that of untreated ones respectively, and reached the peak at 9 h under low temperature, 7.03 fold higher than that of the control. In addition, from qRT-PCR results, *MbMYBC1* was more sensitive to cold and drought stimuli.

### Increased tolerance of transgenic *A. thaliana* to low temperature stress

**Figure 5:**
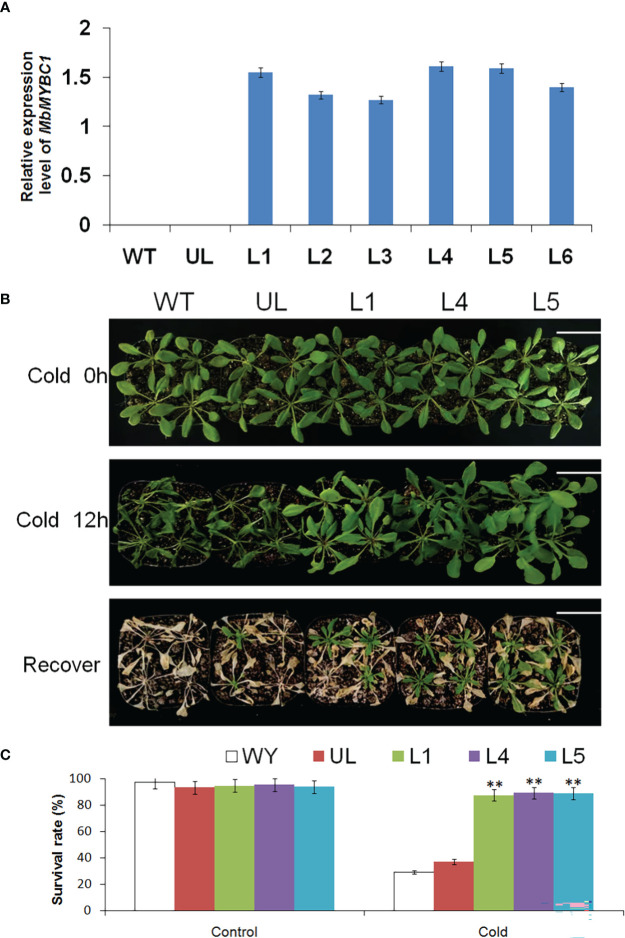
Phenotype and survival rate of *A. thaliana* under low temperature stress. **(A)** The relative expression of *MbMYBC1* in WT, UL and transgenic *A. thaliana*. **(B)** The phenotypes of WT, UL, L1/4/5 lines after treatment at -4°C for 0 h, 12 h and recovery growth. **(C)** The survival rate of *A. thaliana* under control and low temperature stress. Bar = 5 cm. (**, *p* ≤ 0.01).

After cold and drought stress treatment on *Arabidopsis*, the qRT-PCR results of *MbMYBC1* in each line were shown in **Figure 5A**, in which WT and UL lines were the controls. In the identified 6 T_2_ generation transformation lines (L1, L2, L3, L4, L5, L6), *MbMYBC1* gene had a higher expression level in L1, L4, L5. Therefore, these 3 lines were used to cultivate T_3_ generation *A. thaliana*.

**Figures 5B, C** showed the phenotypic changes and survival of *A. thaliana* after low temperature stress. Without treatment, all *Arabidopsis* grew well, showing a healthy state. The temperature of its growth environment was set at -4°C. After 12 h of growth, the leaves of WT and UL lines became soft and shrunk, and the plants were short. The transgenic lines suffered less damage, and the changes were not very obvious. After the normal growth temperature was restored, WT and UL almost all dead, and the survival rate was only 29.3, 37.2. However, some transgenic lines still survived, with the survival rates of 87.5, 89.1 and 88.8 respectively. These results indicated that *MbMYBC1* played a significant role in improving the cold resistance of *A. thaliana*.

**Figure 6:**
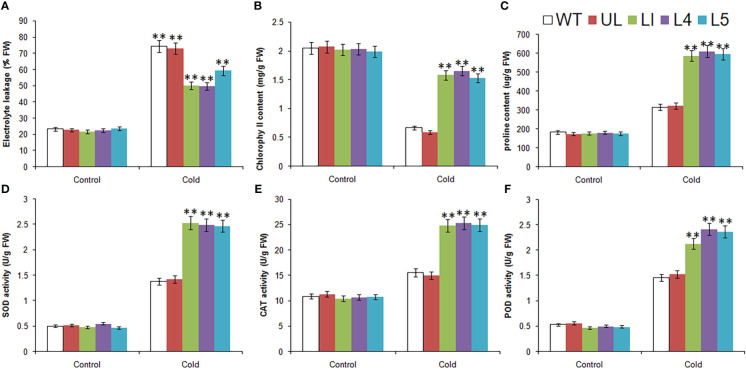
Physiological and biochemical indicatorsin *MbMYBC1*-OE *A. thaliana*. **(A)** EL, **(B)** Chlorophyll content, **(C)** proline content, **(D)** SOD activities, **(E)** CAT activities, and **(F)** POD activities. (**, *P* ≤0.01).The control was the index in the WT. All data were the average of 3 measurements.

Without treatment, the related physiological and biochemical indexes of all plants were roughly in the same level. After low temperature stress, there were obvious changes, which can be seen from **Figure 6**. The EL, content of proline and the activities of SOD, CAT and POD were increased in all *Arabidopsis*, but the increase was greater in L1/4/5, indicating that the degree of cytoplasmic membrane peroxidation in transgenic lines was relatively low. Chlorophyll content of all lines decreased, but the decrease was greater in WT and UL lines, indicating that they suffered more severe stress and chloroplast structure was severely damaged. It can be inferred that *MbMYBC1* can effectively improve the cold tolerance of plants.

### Expression analysis of cold resistant downstream genes in *MbMYBC1*-OE *A. thaliana*

**Figure 7:**
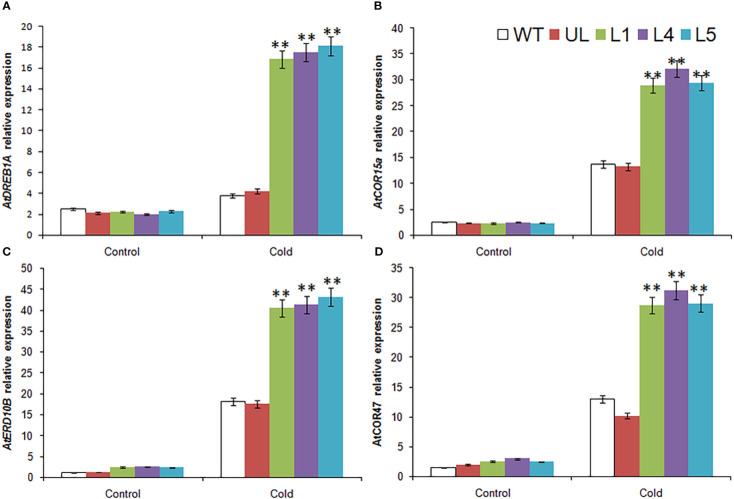
Expression of 4 cold stress related genes in *A. thaliana*. The expression leave of **(A)** *AtDREB1A*, **(B)** *AtCOR15a*, **(C)** *AtERD10B* and **(D)** *AtCOR47*. (**, *P* ≤0.01). The control was the index in the WT. All data were the average of 3 measurements.

The expression of four cold stress related target genes downstream of *MbMYBC1* was detected by qRT-PCR. The results showed that in **Figure 7**, these 4 genes were *AtDREB1A*, *AtCOR15a*, *AtERD10B* and *AtCOR47*. Without -4°C treatment, the expression levels of these genes in all *Arabidopsis* were close and very low. After cold stress, their expression increased significantly, especially in *MbMYBC1*-OE *A. thaliana*, which was much higher than that in UL and WT lines. Therefore, *MbMYBC1* may improve the adaptability of plants to low temperature through positive regulation of downstream related target genes.

### Increased tolerance of transgenic *A. thaliana* to drought stress

**Figure 8:**
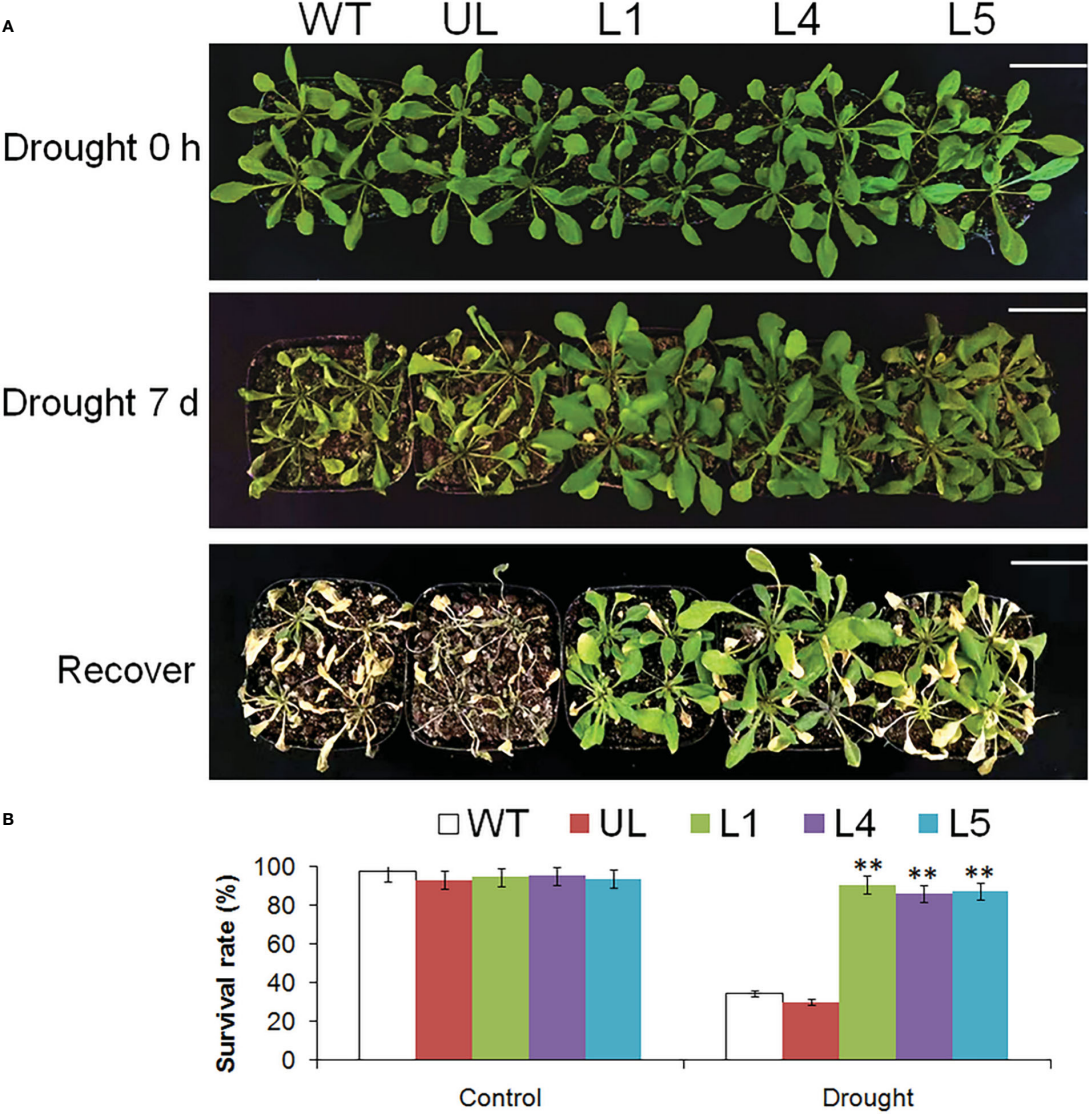
Phenotype and survival rate of *A. thaliana*. **(A)** The phenotypes of WT, UL, L1/4/5 lines were observed after 0 and 7 d of stopping watering and recovering growth. **(B)** The survival rate of *A. thaliana* under control and drought stress. Bar= 5 cm. (**, *p* ≤ 0.01).

**Figure 8** showed the phenotype changes and survival rate of *Arabidopsis* after drought stress. When growing in a good environment, the appearance of all plants was basically the same, and the leaves were plump. After 7 days of stopping watering, the phenotypes of WT and UL lines had changed significantly, the leaves became smaller and curly, while the changes of transgenic plants were not very obvious. After 3 days of normal watering, only 34.5% and 30.2% of WT and UL survived, while the survival rates of L1, L4 and L5 were 90.7%, 86.4% and 87.4% respectively.

**Figure 9:**
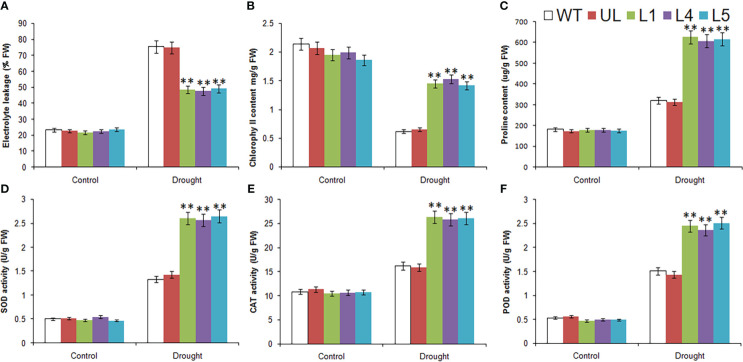
Physiological and biochemical indicatorsin *MbMYBC1*-OE *A. thaliana* under drought stress. **(A)** EL, **(B)** Chlorophyll content, **(C)** proline content, **(D)** SOD activities, **(E)** CAT activities, and **(F)** POD activities. (**, *P* ≤0.01). The control was the index in the WT. All data were the average of 3 measurements.

Under the control condition, the determination results of relevant physiological and biochemical indexes of WT, UL and L1/4/5 lines were basically the same (**Figure 9**). After drought stress, except for the reduction of chlorophyll content, EL, proline content, SOD, CAT and POD activities were higher than those in the control group, and these indicators were significantly higher in transgenic lines than in WT and UL lines. As for chlorophyll content, it was also higher in transgenic plants, it can be preliminarily inferred that overexpression of *MbMYBC1* is helpful to improve the survival rate of plants in arid environments.

### Expression analysis of drought resistant downstream genes in *MbMYBC1*-OE *A. thaliana*

**Figure 10:**
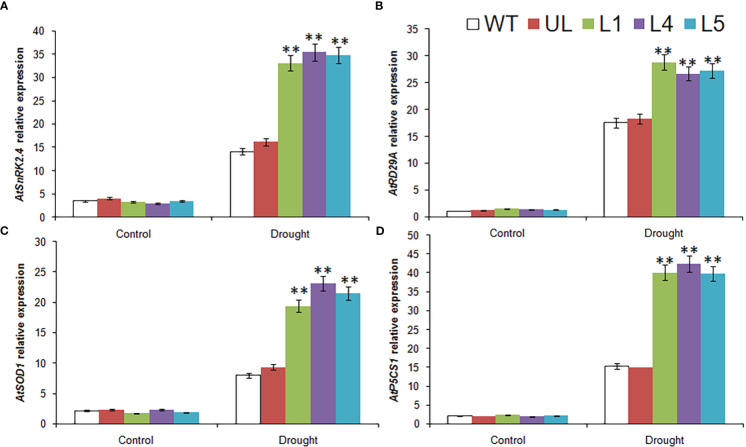
Expression of 4drought stress related genes in *A. thaliana*. The expression leave of **(A)** *AtSnRK2.4*, **(B)** *AtRD29A*, **(C)** *AtSOD1 and* **(D)** *AtP5CS1*. (**, *P* ≤0.01). The control was the index in the WT. All data were the average of 3 measurements.

In order to further explore the molecular mechanism of plant drought resistance, the expression levels of several related downstream target genes were detected by qPCR in this study. The results were shown in **Figure 10**. These 4 genes were *AtSnRK2.4*, *AtRD29A*, *AtSOD1* and *AtP5CS1*, respectively. Before watering was stopped, the expression levels of these 4 genes in all plants were at the same low level. After water shortage treatment, their expression levels in all lines were improved to varying degrees, especially in transgenic lines (L1, L4, L5), although they were also increased in WT and UL lines, but the expression level was much lower than that in L1, L4 and L5.

## Discussion

Because of the adverse changes in the environment, the fixed plants will inevitably suffer from various abiotic stresses, which will restrict the yield and geographical distribution of horticultural crops, as well as economic development (Zhu, 2016; Han et al., 2018c; Tripathi et al., 2022). In order to cope with these environmental factors threatening survival, plants have evolved a variety of adaptive mechanisms, including morphological, physiological and molecular aspects (Yan et al., 2014; Xiong et al., 2020; Liang et al., 2022a). TFs can play a regulatory role at the transcriptional level, enabling plants to adapt to external environmental stress by changing their own structure or metabolic process (Serrano et al., 2014; Qin et al., 2015; Zhang et al., 2018). It can also increase or inhibit the expression of downstream genes by combining with cis acting elements of target genes, so as to improve plant stress resistance (Liu et al., 2019; Liang et al., 2022b).

As one of the largest TF families, MYB has been proved to be involved in the response of plants to cold and drought stress. Dossa et al. (2019) found that the overexpression of *SiMYB75* can promote the accumulation of ABA in *Arabidopsis*, and promote the up regulation of drought stress related genes in its downstream through ABA dependent pathway, so as to improve the tolerance of *Arabidopsis* to drought stress. Studies have shown that *GmMYB118* can up regulate the expression of genes related to drought stress through ABA signal pathway to enhance the viability of transgenic *Arabidopsis* under drought conditions (Du et al., 2018). Overexpression of *GmMYBJ1* can change some physiological traits of plants to adapt to low temperature environment, and can activate the expression of downstream *AtRD29B*, *AtCOR47*, *AtCOR78*, *AtP5CS* and *AtCOR15a*, which further enhanced the ability of plants to respond to chill environment (Su et al., 2014).

In our experiment, we cloned and separated *MbMYBC1* from *M. baccata* and characterized it. Sequence analysis and cluster analysis of MbMYBC1 and other MYB proteins showed that they may have similar functions and properties, and the highest homology between MbMYBC1 and MsMYBC1 (**Figure 1**). The predicted domain and tertiary structure of MbMYBC1 protein also conformed to the function of the proposed transcription factor (**Figure 2**). The subcellular localization results confirmed that it was a nuclear protein, which was the same as other known MYB proteins (**Figure 3**).

After qPCR detection of *MbMYBC1*, it was found that it could be expressed in young leaves, mature leaves, root and stem, but the expression amount was different (**Figure 4**). Its expression level in young leaves and roots was higher, indicating that the expression of *MbMYBC1* was tissue specific. After detecting the expression of *MbMYBC1* in young leaves and roots under different conditions, it was found that the expression of this gene could be subjected to cold, drought, salt and high temperature stresses, and the expression level changed with time, showing a trend of first rising and then declining. According to this result, we can know that its expression was also stage specific. In addition, we also found that the expression of *MbMYBC1* was more susceptible to the induction of low temperature and drought. In view of this phenomenon, we speculated that *MbMYBC1* may play a key role in regulating the response of plants to low temperature and drought stress.

In order to explore the role of *MbMYBC1* in plant cold and drought resistance, we detected the changes of phenotypic, physiological and biochemical indicators of transgenic *Arabidopsis* after cold and drought stress. Plant phenotype is closely related to damage degree (Singh et al., 2021). Cell membrane is the protective barrier of cells, and the MDA content can reflect the permeability of cell membrane under stress (Jungklang et al., 2017). The harsh environment will make plant cells produce excessive ROS, which will cause serious damage to the structural and functional integrity of chloroplasts, affect the synthesis of chlorophyll, and change the activity of antioxidant enzymes (Kavian et al., 2022; Han et al., 2023). An obvious fact was that WT and UL plants grew weaker after stress treatment. However, transgenic lines still had a high survival rate (**Figures 5**, **Figure 8**). Moreover, EL, content of chlorophyll and proline, activities of SOD, POD and CAT have changed correspondingly in response to stimuli to adapt to cold and drought (**Figures 6**, **Figure 9**). This showed that overexpression of *MbMYBC1* can reduce plant damage under low temperature and drought stress.

**Figure 11:**
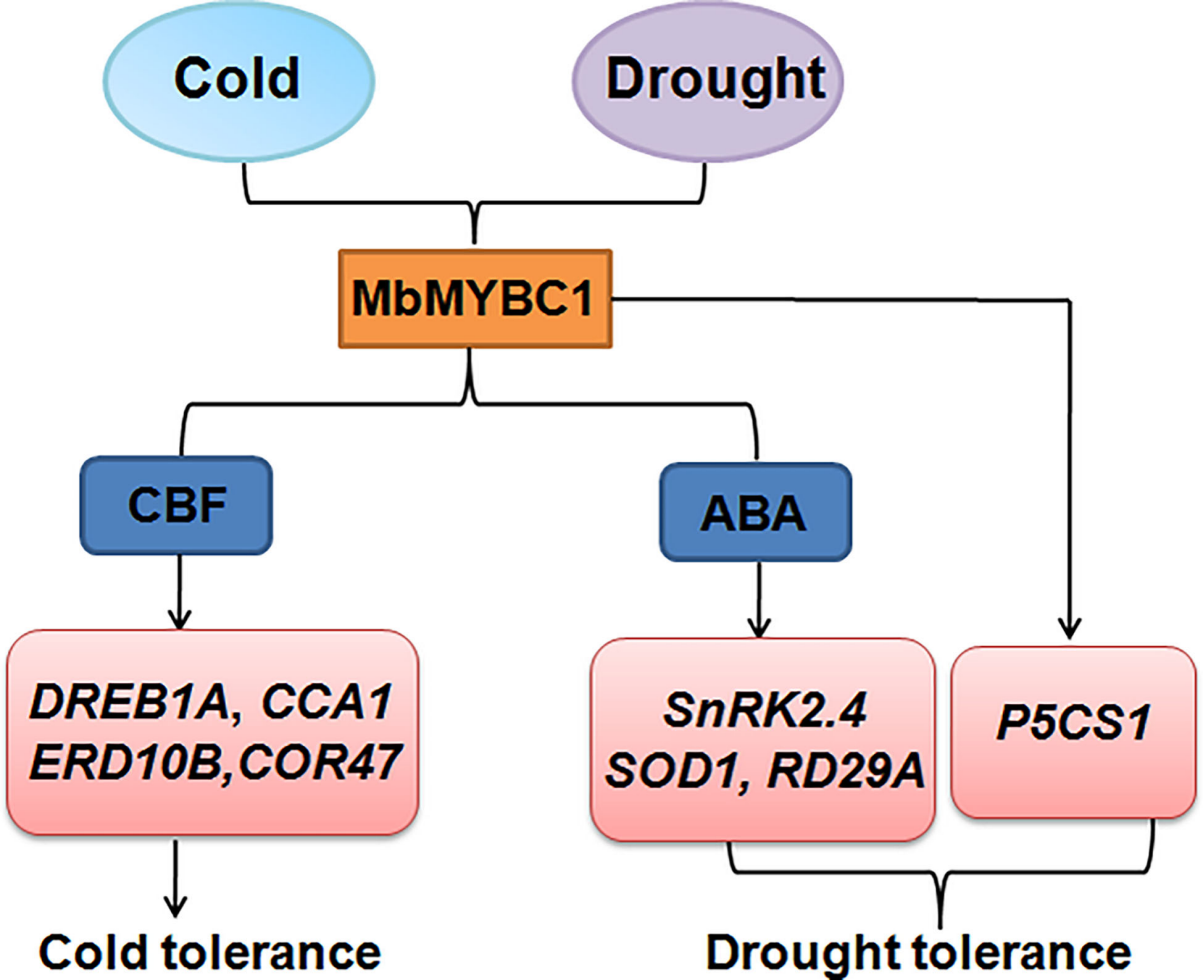
A potential model of *MbMYBC1* regulating plant responses to low temperature and drought stress.

Overexpression of *MbMYBC1* also activated the expression of downstream related target genes (**Figures 7**, **10**). Therefore, based on this result and the proven conclusion, we predicted the possible pathway of this gene in response to cold and drought stress, as show in **Figure 11**. When subjected to low temperature stress, *MbMYBC1* first perceives cold signals and activates the expression of downstream cold response genes *DREB1A*, *CCA1*, *ERD10B* and *COR47* through CBF pathway, thus endowing plants with cold tolerance. After receiving drought stimulation, *MbMYBC1* transmits signals to downstream *SnRK2.4*, *RD29A*, *SOD1* containing ABA response elements through ABA dependent pathway to promote their expression, and can also directly activate the expression of *P5CS1*, giving plants drought tolerance.

## Conclusion

We isolated and characterized a new MYB gene, *MbMYBC1*, which encodes a protein localized in the nucleus. Overexpression of *MbMYBC1* can change the relevant physiological and biochemical indicators and also regulate the expression of stress-related genes so as to improve the survival ability of plants under cold and drought conditions. This provides a theoretical basis and candidate genes for improving apple quality.

## Data availability statement

The original contributions presented in the study are included in the article/**Supplementary Material**. Further inquiries can be directed to the corresponding authors.

## Author contributions

WL contributed to the conception of the study, TW, YW, and XL performed the experiment, contributed significantly to analysis and manuscript preparation, JH and DH and performed the data analyses and wrote the manuscript. All authors contributed to the article and approved the submitted version.

## Supplementary material

Supplementary Figure 1 Gene sequence and amino acid sequence of MbMYBC1. The underscore marked the conservative sequence of MYB.
