# Supplementary material for: MbMYBC1, a M. baccata MYB transcription factor, contribute to cold and drought stress tolerance in transgenic Arabidopsis
Source: Front Plant Sci. 2023 Feb 16;14:1141446. doi: 10.3389/fpls.2023.1141446 (PMC9978498; doi:10.3389/fpls.2023.1141446)
Supplement: Supplementary Figure 1 — Gene sequence and amino acid sequence of MbMYBC1. The underscore marked the conservative sequence of MYB. [file DataSheet_1.zip › Supplementary materials/Tables.docx]

**Table S1.** Primers used in this study

| **Primer Name** | **Primer sequence (5'→3')** | **Function** |
| --- | --- | --- |
| *MbMYBC1*-F | ATGAGGGAAGATGATGAGTCG | Clone full length of *MbMYBC1* |
| *MbMYBC1*-R | CAATCATCCCCAGTTGGAAAT | Clone full length of *MbMYBC1* |
| *Actin*-F | ACACGGGGAGGTAGTGACAA | q-PCR for *Actin* |
| *Actin*-R | CCTCCAATGGATCCTCGTTA | q-PCR for *Actin* |
| *MbMYBC1*-qF | TAGAACGGGAGCGGAAACTG | q-PCR for *MbMYBC1* |
| *MbMYBC1*-qR | AAGGTTCAGGAGATGTATCGGACT | q-PCR for *MbMYBC1* |
| *AtDREB1A-F* | ATGAACTCATTTTCTGCTTTTTCTG | Clone full length of *AtDREB14* |
| *AtDREB1A-R* | TTAATAACTCCATAACGATACG | Clone full length of *AtDREB14* |
| *AtCCA1-F* | ATGGAGACAAATTCGTCTGG | Clone full length of *AtCCA1* |
| *AtCCA1-R* | TCATGTGGAAGCTTGAGTTTCC | Clone full length of *AtCCA1* |
| *AtERD10B*-F | GCAGCAGGAGGAGAAGGG | Clone full length of *AtERD10B* |
| *AtERD10B*-R | CACCAGGAAGAAGCCCATC | Clone full length of *AtERD10B* |
| *AtCOR47*-F | TGGTTGTAACGGAGCATC | Clone full length of *AtCOR47* |
| *AtCOR47*-R | CCCCAAGAAATCAAACAA | Clone full length of *AtCOR47* |
| *AtSnRK2.4*-F | GAGGAAATGGGGATGCAGAT | Clone full length of *AtSnRK2.4* |
| *AtSnRK2.4*-R | TTCTCACTTCTCCACTTGCG | Clone full length of *AtSnRK2.4* |
| *AtCOR15a*-F | CAACAGAGGAATCACCAGCGA | Clone full length of *AtCOR15a* |
| *AtCOR15a*-R | CTCTGCTGTCTTGTCGTGGTGT | Clone full length of *AtCOR15a* |
| *AtRD29A*-F | CAACGAGGGGAAGATAAAAGTGT | Clone full length of *AtRD29A* |
| *AtRD29A*-R | AGCCAGATGATTTTGGAGCCT | Clone full length of *AtRD29A* |
| *AtP5CS1*-F | GATACGGATATGGCAAAGCG | Clone full length of *AtP5CS1* |
| *AtP5CS1*-R | CCAAGTCCAAATCGGAAACC | Clone full length of *AtP5CS1* |
|  |  |  |

**Table 2.** The components of cloning *MbMYBC1*

| Component | Volume |
| --- | --- |
| cDNA  *MbMYBC1*-qF/R  2×Flash Hot Start MasterMix (Dye)  dd H2O  Total Volume | 1.5 μl  1.0 μl  12.5 μl  9.0 μl  25.0 μl |
