# Supplementary figures and images for: MbMYBC1, a M. baccata MYB transcription factor, contribute to cold and drought stress tolerance in transgenic Arabidopsis
Source: Front Plant Sci. 2023 Feb 16;14:1141446. doi: 10.3389/fpls.2023.1141446 (PMC9978498; doi:10.3389/fpls.2023.1141446)

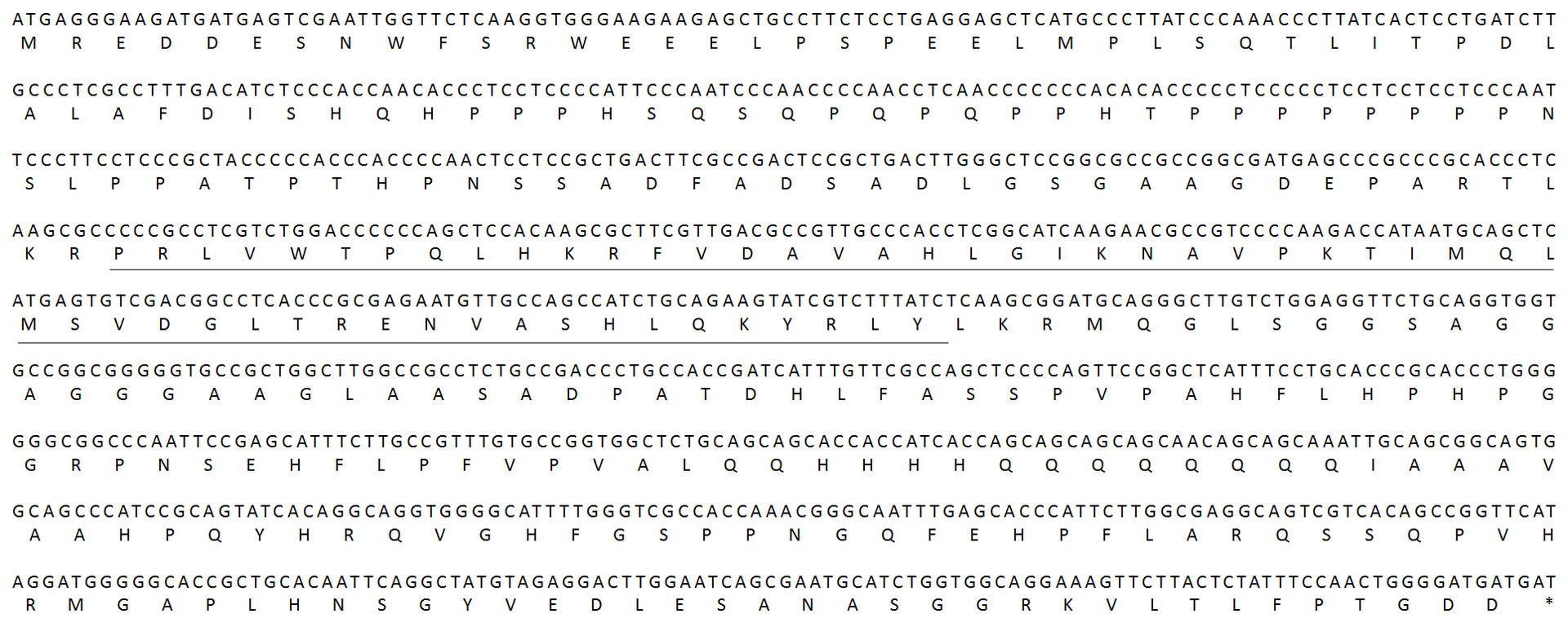

Supplement: Supplementary Figure 1 — Gene sequence and amino acid sequence of MbMYBC1. The underscore marked the conservative sequence of MYB. [file DataSheet_1.zip › Supplementary materials/Figure S1.tif]
